# Enhancing Monitoring Performance: A Microservices Approach to Monitoring with Spyware Techniques and Prediction Models

**DOI:** 10.3390/s24134212

**Published:** 2024-06-28

**Authors:** Anubis Graciela de Moraes Rossetto, Darlan Noetzold, Luis Augusto Silva, Valderi Reis Quietinho Leithardt

**Affiliations:** 1 Federal Institute of Education, Science and Technology Sul-Rio-Grandense, Passo Fundo 99.064-440, RS, Brazil; anubisrossetto@ifsul.edu.br (A.G.d.M.R.); darlannoetzold.pf149@academico.ifsul.edu.br (D.N.); 2 Department of Computer Science and Automation, University of Salamanca, 37008 Salamanca, Spain; 3 Lisbon School of Engineering (ISEL), Polytechnic University of Lisbon (IPL), 1549-020 Lisbon, Portugal; valderi.leithardt@isel.pt; 4 Center of Technology and Systems (UNINOVA-CTS) and Associated Lab of Intelligent Systems (LASI), 2829-516 Caparica, Portugal

**Keywords:** data leakage, electronic monitoring, hate speech, microservices, prediction

## Abstract

In today’s digital landscape, organizations face significant challenges, including sensitive data leaks and the proliferation of hate speech, both of which can lead to severe consequences such as financial losses, reputational damage, and psychological impacts on employees. This work considers a comprehensive solution using a microservices architecture to monitor computer usage within organizations effectively. The approach incorporates spyware techniques to capture data from employee computers and a web application for alert management. The system detects data leaks, suspicious behaviors, and hate speech through efficient data capture and predictive modeling. Therefore, this paper presents a comparative performance analysis between Spring Boot and Quarkus, focusing on objective metrics and quantitative statistics. By utilizing recognized tools and benchmarks in the computer science community, the study provides an in-depth understanding of the performance differences between these two platforms. The implementation of Quarkus over Spring Boot demonstrated substantial improvements: memory usage was reduced by up to 80% and CPU usage by 95%, and system uptime decreased by 119%. This solution offers a robust framework for enhancing organizational security and mitigating potential threats through proactive monitoring and predictive analysis while also guiding developers and software architects in making informed technological choices.

## 1. Introduction

In recent years, the development of corporate applications based on microservices has become a prevalent approach in the software industry. In this context, frameworks like Spring Boot and Quarkus have become prominent choices to simplify and accelerate development. However, choosing between these frameworks is not trivial; it involves carefully considering performance, efficiency, and scalability factors.

The primary challenge addressed in this paper is the lack of comprehensive, data-driven comparisons between Spring Boot and Quarkus that can guide developers in making informed decisions. With the increasing complexity of modern systems, there is an urgent need to understand how these frameworks perform under various conditions, specifically in terms of startup time, memory usage, throughput, and scalability.

This article proposes a comparative performance analysis between Spring Boot and Quarkus, focusing on objective metrics and quantitative statistics. The goal is to provide an in-depth understanding of the performance differences between these two platforms, using various tools and benchmarks recognized in the computer science community.

This analysis is important because developers and software architects need objective guidance when choosing an application framework. With modern systems’ increasing complexity, resource consumption efficiency and the ability to scale horizontally become crucial criteria. Moreover, optimizing the development time is vital to remaining competitive in the dynamic software market.

This article will explore metrics such as startup time, memory usage, throughput, and scalability using established benchmarks like TechEmpower and specific profiling tools. Furthermore, we will support our conclusions with bibliographic references highlighting the importance of informed and data-based technological choices to optimize the development lifecycle and operational efficiency.

The urgency to adopt architectural approaches that facilitate scalability and maintenance in microservices environments is highlighted by [1]. Additionally, Ref. [2] emphasizes the importance of efficient frameworks in ensuring the long-term viability of these architectures, underlining the critical relevance of informed technological choices.

Specifically regarding Spring Boot, the research conducted by [3] offers significant insights into the features and benefits of this framework. Moreover, the study addresses the challenges inherent in the extensive use of annotations in Spring applications, providing a comprehensive perspective on the complexity of development in microservices-based environments.

Concerning Quarkus, research like that of [4] explores energy efficiency in Java applications, presenting a unique view of resource consumption in microservices environments. These detailed analyses contribute to a holistic understanding of the performance implications associated with Quarkus, informing our comparative approach.

These studies enrich our theoretical understanding and provide a solid foundation for the proposed comparative analysis. It is emphasized that all analyses and tests were conducted on a complex application involving extensive and complex data and intricate relationships between various tables. By contextualizing performance metrics within this challenging environment, our study gains relevance by contributing to understanding the trends and challenges faced in developing microservices-based applications. Thus, these bibliographic references further strengthen the foundation of our study.

## 2. Background

This chapter will detail the technologies used in the application’s development, the testing tools adopted, and the fundamental concepts applied in evaluating the results. It offers a comprehensive view of the literature, organizing the information into four main sections: frameworks used, employed integrations, selected testing tools, and essential concepts for analyzing the results.

### 2.1. Frameworks

#### 2.1.1. Spring Boot

Spring Boot, known for simplifying the development of Java applications, brings a series of benefits and challenges concerning performance. Its automatic configuration and “starters” provide agility at the project’s start, reducing the need for extensive configuration coding. This boosts productivity, allowing developers to focus on the app’s functionalities instead of worrying about configuration details.

One of the primary reasons for using Spring Boot is its extensive ecosystem and community support. Spring Boot integrates seamlessly with a wide range of other Spring projects, offering comprehensive solutions for various application needs, from data access and security to messaging and cloud deployment. This integration capability allows developers to leverage robust tools and libraries, ensuring the application evolves and scales effectively as requirements change. The strong community support also provides a wealth of resources, documentation, and third-party extensions, which can significantly accelerate development and troubleshooting processes.

However, this automation can result in performance challenges. Increased memory consumption is one of them, as automatic configuration can load various modules and resources, generating overhead in memory allocation. In complex applications, the startup can be slower due to extensive configuration and classpath analysis, affecting the application’s startup time.

Hidden complexity is a delicate point: although simplification is an advantage, hidden complexity can make it difficult to identify performance bottlenecks. Issues relating to the framework’s configuration can be challenging to find and resolve.

Another aspect is the size of the generated artefact. By automatically including libraries and modules, Spring Boot can result in more significant artefacts. This affects deployment time and the server’s resource consumption, directly impacting performance in cases of limited infrastructure.

To address these challenges, it is crucial to profile the application, identifying areas for improvement and performance bottlenecks. Selectively optimizing specific application parts is essential, avoiding including unused features and adjusting the automatic startup of modules. Additionally, keeping frequent updates of Spring Boot and continuously monitoring the application’s performance are fundamental practices to mitigate possible impacts on performance.

#### 2.1.2. Quarkus

Quarkus is an innovative framework for developing native Java applications in the cloud. It offers a distinct set of performance benefits and challenges. Its architecture and purpose are shaped to optimize application performance and efficiency, especially in cloud and container environments.

A key reason for adopting Quarkus is its strong focus on Kubernetes-native development. Quarkus is built to integrate seamlessly with Kubernetes, offering features like live coding, streamlined container image generation, and comprehensive support for cloud-native patterns. This makes it an ideal choice for developing applications meant to run in containerized environments, where rapid scaling and efficient resource utilization are crucial. By aligning closely with Kubernetes, Quarkus simplifies the deployment and management of applications in cloud-native infrastructures, enhancing the developer experience and operational efficiency.

One of the main benefits of Quarkus is its approach to creating native applications, enabling shorter startup times and lower memory consumption compared with traditional applications. This is achieved through ahead-of-time compilation, quick loading, and the ability to package only the necessary parts of the application.

However, despite these advantages, some challenges can arise when working with Quarkus. The complexity of the ahead-of-time compilation process can make building and development more intricate, especially for those accustomed to the traditional development paradigm. Moreover, certain Quarkus features may introduce limitations on some functionalities or require careful adaptation of development practices.

The optimized nature of Quarkus, while a significant positive point, may also require careful planning to ensure that its optimization does not sacrifice the flexibility or scalability of the application. Specific strategies to balance performance and development flexibility are necessary to leverage the benefits offered by Quarkus fully [5].

Quarkus’s continuous evolution, introducing new features and improvements, represents significant potential for modern and highly efficient applications. However, to maximize its benefits, developers must deeply understand the framework’s characteristics and apply development strategies that maximize its performance potential.

### 2.2. Integrations

This chapter summarizes the integrations used in both applications discussed in this article:

1. PostgreSQL: An open-source relational Database Management System (DBMS) known for its scalability, support for transactional integrity, Multi-Version Concurrency Control (MVCC), stored procedures, and triggers. It stands out for its extensibility, robustness, and Structured Query Language (SQL) standards compatibility, backed by an active community [6].
2. Redis: An open-source, low-latency, high-performance caching system supporting various structured data types in a key-value structure. It is chosen for its scalability, efficiency, and ease of use across various applications [7].
3. FlywayDB: An open-source tool for database schema management and versioning, operating under the principle of “migrations as code”. It facilitates the automation of migrations in agile development and Development and Operations (DevOps) environments [8].
4. RabbitMQ: A messaging platform that implements Advanced Message Queuing Protocol (AMQP), offering modular architecture and features like message queues. Evaluations demonstrate its performance and scalability in various scenarios [9].
5. Keycloak: An identity and access management platform offering robust authentication and authorization, supporting various authentication methods and modern standards, facilitating security in applications [10,11].
6. Prometheus and Grafana: Tools for system and application monitoring, with Prometheus focused on metrics collection and Grafana on visualization. Their integration enables effective monitoring and interactive visualization [12,13,14,15].
7. Docker: A platform that facilitates the creation, deployment, and running of container applications, promoting portability and efficiency. Complemented by tools like Docker Compose and Docker Swarm, it facilitates container automation and orchestration [16,17,18,19,20].

### 2.3. Tools

In this chapter, we describe the tools used to test and evaluate the performance of the work developed:

Postman: An Application Programming Interface (API) development tool that facilitates testing, documentation, and collaboration in API creation. It allows developers to send Hypertext Transfer Protocol (HTTP) requests, verify responses, generate interactive documentation, and collaborate in teams, positively impacting the API development community [21]. JMeter: An Apache performance testing tool to assess web applications under load. It offers features to simulate real scenarios, generate load on servers, collect detailed performance metrics, and identify bottlenecks, and is widely recognized for its efficiency and flexibility [22,23,24]. Custom test application: Specifically developed for this work, this application enables sending bulk requests in parallel and with varied content sizes and conducting security tests to ensure data integrity [25].

### 2.4. Analysis Concepts

Statistical calculations were employed to validate the test results and conduct more detailed analyses of the application’s performance. These calculations map the relationship between monitored attributes, determine the application’s capacity according to the used hardware, and identify possible performance bottlenecks.

In this sense, this section covers the following statistical calculations: correlation coefficient, regression analysis, load curve, and response time analysis.

#### 2.4.1. Correlation Coefficient

The correlation coefficient is a widely used statistical measure to assess the relationship between two variables. It measures the degree of linear association between the variables, ranging from −1 to 1.

According to [26], one of the most common ways to calculate the correlation coefficient is the Pearson correlation coefficient, represented by *r*. The formula for the Pearson correlation coefficient is given by

(1) $$r=\frac{\sum_{i=1}^{n}\left(x_{i}-\bar{x}\right)\left(y_{i}-\bar{y}\right)}{\sqrt{\sum_{i=1}^{n}{\left(x_{i}-\bar{x}\right)}^{2}\sum_{i=1}^{n}{\left(y_{i}-\bar{y}\right)}^{2}}}$$

where $x_{i}$ and $y_{i}$ are the values of variables *x* and *y* for each observation, $\bar{x}$ and $\bar{y}$ are the averages of *x* and *y* values, respectively, and *n* is the number of observations [26].

It is important to highlight that the Pearson correlation coefficient is only suitable for measuring the linear relationship between the variables. This coefficient may not capture other forms of non-linear association.

Other correlation measures can be used in different contexts. For example, the Spearman correlation coefficient is a non-parametric measure assessing the variables’ monotonic relationship. It is calculated based on the rankings of the variables’ values [27].

#### 2.4.2. Regression Analysis

Regression analysis is a statistical technique for studying the relationship between a dependent variable and one or more independent variables. It seeks to model this relationship through a linear equation.

One of the most common methods to perform regression analysis is the least-squares method. This method finds the coefficients of the linear equation that minimize the sum of the squares of the differences between the observed values and the values predicted by the model [28].

The equation of simple linear regression can be represented by

(2) $$y=\beta_{0}+\beta_{1}x+\varepsilon$$

where *y* is the dependent variable, *x* is the independent variable, $\beta_{0}$ is the intercept, $\beta_{1}$ is the regression coefficient, and ε is the error term [29].

Different regression analysis techniques exist, such as simple linear regression, multiple linear regression, and non-linear regression. Each of these techniques has its assumptions and methods of evaluation [29].

Simple Linear Regression: A method that models the relationship between two dependent and independent variables through a linear equation. The basic equation is $y=\beta_{0}+\beta_{1}x+\varepsilon$, where *y* is the dependent variable, *x* is the independent variable, $\beta_{0}$ is the intercept, $\beta_{1}$ is the regression coefficient, and ε is the error term. The least-squares method is often used to find the coefficients that minimize the sum of the squares of the differences between the observed and predicted values [30].

Multiple Linear Regression: Extends the idea of simple linear regression to more than one independent variable. The equation becomes $y=\beta_{0}+\beta_{1}x_{1}+\beta_{2}x_{2}+\ldots +\beta_{p}x_{p}+\varepsilon$, where $x_{1},x_{2},\ldots ,x_{p}$ are the independent variables and $\beta_{0},\beta_{1},\beta_{2},\ldots ,\beta_{p}$ are the regression coefficients associated with each independent variable [31].

Non-Linear Regression: While linear regression relies on linear equations, non-linear regression allows the model to fit more complex relationships between variables. This can be performed using non-linear functions, such as exponential or logarithmic, to describe the relationship between the variables [32].

Exponential and Logarithmic Regression: These are types of non-linear regression. Exponential regression models relationships where the data fit an exponential curve, while logarithmic regression models relationships that fit a logarithmic curve [33].

Power Series: This is a form of non-linear regression where the model fits a series of polynomial terms rather than a single equation. This allows for modeling complex relationships that a single linear function cannot represent [34].

Each type of regression analysis has its applications and underlying assumptions. The choice of method depends on the nature of the data and the expected relationship between the involved variables. Validation and interpretation of the results are also crucial to ensure that the model is appropriate and helpful in making predictions or inferences.

#### 2.4.3. Load Curve

The load curve is a graphical representation of energy consumption over time. It is often used to analyze the consumption profile of a particular load or system.

Resource consumption data must be collected regularly to calculate the load curve. These data can also be used to estimate consumption when measurements are unavailable. A commonly used approach is the interpolation method, which consists of filling in missing values using a function that fits the available data.

According to [35], the general formula of an *n*-degree polynomial used to interpolate a load curve is given by

(3) $$f\left(t\right)=a_{0}+a_{1}t+a_{2}t^{2}+\ldots +a_{n}t^{n}$$

where f(t) is the estimated value of the load curve at time *t*, and $a_{0},a_{1},\ldots ,a_{n}$ are the polynomial coefficients.

Other methods and statistical models can be used to calculate the load curve, such as non-linear regression and time series [35].

#### 2.4.4. Response Time Analysis

Response time analysis is a technique used to evaluate an application’s performance regarding the time needed to respond to user requests. It is particularly relevant in web applications, where response speed is critical to user experience [36].

One of the main indicators used in response time analysis is the average response time. It is calculated as the average response times of a set of requests. The formula for average response time is given by

(4) $$\mathrm{Average}\;\mathrm{Response}\;\mathrm{Time}=\frac{\sum_{i=1}^{n}t_{i}}{n}$$

$t_{i}$ is the response time of the *i*-th request, and *n* is the total number of requests [37].

## 3. Related Works

### 3.1. Framework Comparison

This section presents a literature review on performance comparison between APIs developed using the Spring and Quarkus frameworks. It discusses the main results from previous studies and the differences between the two frameworks that might influence their performance.

Various studies in the literature have compared the performance of Spring Boot and Quarkus applications in API scenarios. Generally, these analyses indicate that Quarkus offers superior performance compared with Spring Boot.

For example, the article “A Performance Comparison of Spring Boot and Quarkus for Microservices” [38] compares the performance of the two frameworks in a microservices application, concluding that Quarkus shows better results in all benchmarks, including startup time, response time, and memory consumption.

Another article, “Spring Boot vs. Quarkus: A Performance Comparison” [5], also addresses performance comparison in a more straightforward application. In this case, Quarkus offers a faster startup, while Spring Boot stands out in response time.

The book “Spring Boot vs. Quarkus: A Comparison of Two Java Frameworks” provides an overview of the main discrepancies between Spring Boot and Quarkus, discussing how these differences can impact the performance of both frameworks.

Another study, “Performance Comparison of Spring Boot and Quarkus: A Case Study” [39], presents a case study comparing the performance of Spring Boot and Quarkus in complex applications, concluding that Quarkus excels in all the evaluated benchmarks.

In the article “Comparative Performance Analysis between Spring Boot and Quarkus: An Empirical Study” by Gabriel Ferreira da Rosa, Kleinner Farias, and Carlos Fernando Santos Xavier [40], a comparative performance analysis between Spring Boot and Quarkus is presented. This study employs a use case involving messaging communication scenarios and their persistence in a database, using CPU, RAM, and message processing time measurements. The results indicate that Quarkus performs slightly better in most tested scenarios, suggesting an advantage for using Quarkus in specific application development contexts.

### 3.2. Monitoring Platforms

The following section analyzes various works on the monitoring platform discussed in this paper. It includes reviewing several tools that share similar objectives with the research presented, providing context and comparison to enhance the understanding of the current landscape in monitoring technology.

Among commercial solutions, Kickidler [41] aims to automate employee management by providing tools for monitoring employee computers and detecting violations during work hours. Key features include real-time screen viewing with multi-user mode, employee work time reports, and a keylogger to save keystroke history. However, the unpaid version has limited functionalities. While Kickidler offers several monitoring features, it does not include internet traffic monitoring, vulnerability alerts, or hate speech alerts.

ActivTrak, another commercial solution, monitors activity, analyzes performance and behavior, and detects insider threats on work computers. Its features include real-time screen viewing (without multi-user mode), time accounting reports, a website blocker, keylogger, and screenshot capture. ActivTrak, however, lacks process monitoring, vulnerability alerts, and hate speech alerts.

FSense [42] monitors computer usage and records access to unauthorized websites and applications, aiming to increase team productivity. It provides a dashboard with graphs and reports, summaries of monitored activities, idleness, and locked machines, and captures screenshots every 30 s for process analysis. Despite its comprehensive monitoring capabilities, FSense does not offer keylogging, process monitoring, vulnerability, or hate speech alerts.

Paschalides et al. [43] propose Mandola, a system for reporting and monitoring online hate speech. It uses an ensemble-based classification algorithm comprising six components that communicate to consume, process, store, and visualize statistical information on online hate speech. Mandola focuses primarily on hate speech detection within browsers and does not provide keylogging, screenshot capture, process monitoring, or vulnerability alerts.

Modha et al. [44] present an approach to detect and visualize aggression on social media. They designed a browser plug-in interface for Facebook and Twitter to visualize aggressive comments posted on users’ timelines, available to the general public and the industry. This solution is limited to browser-based hate speech alerts and lacks keylogging, screenshot capture, process monitoring, internet traffic monitoring, and vulnerability alerts.

DeskTime [45] and StaffCop are productivity control solutions, known as “Bossware”, used to measure employee efficiency without a focus on security or mitigating hate speech. These solutions do not include keylogging, screenshot capture, process monitoring, internet traffic monitoring, vulnerability alerts, or hate speech alerts.

Various studies have consistently shown that Quarkus outperforms Spring Boot in key metrics such as startup time, response time, and memory consumption. However, these studies often lack a detailed examination of specific aspects addressed in this article. Notably, this work includes an extensive evaluation of process monitoring, internet traffic monitoring, and the inclusion of advanced security features such as vulnerability alerts and hate speech detection, which are not thoroughly covered in existing literature. These additional metrics provide a more holistic view of framework performance in real-world scenarios, particularly in environments that require robust security and monitoring capabilities. The review of commercial solutions further emphasizes the importance of selecting tools offering comprehensive features tailored to organizational needs. This comparative analysis underscores the strengths and weaknesses of each solution, providing a valuable reference point for future research and practical application in microservices and API development.

## 4. Developed Applications

Both applications are identical regarding their functionalities, classes, services, endpoints, and objectives; the only difference is the framework used and some configurations. Thus, this chapter will treat the application as a single entity, clarifying the architecture and the developed points.

**Figure 1:**
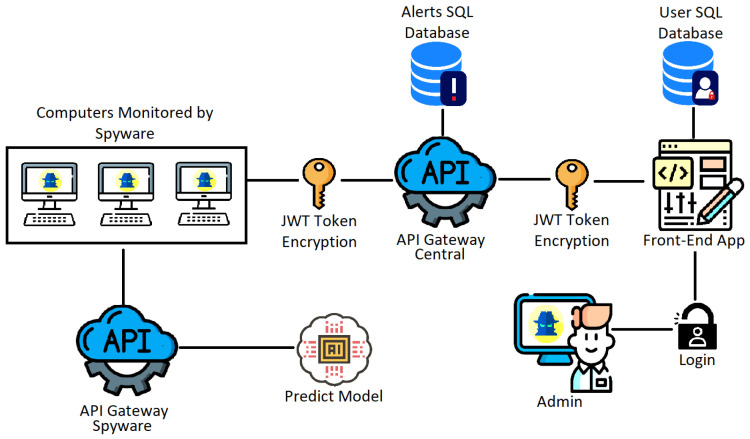
Architecture.

This application aims to use Spyware techniques to monitor corporate or institutional computers, using prediction models to detect hate speech and monitor network packets, vulnerabilities, and malicious processes. As seen in Figure 1, the proposed architecture for this solution involves several applications, which require a significant amount of information exchange, demanding good performance from the Central API Gateway. The architecture includes components for JWT token encryption to ensure secure communication, SQL databases for storing alerts and user information, and a front-end application for user interaction. Additionally, integrating a predictive model enhances the system’s capability to proactively identify threats, making the overall system robust and efficient in handling security tasks. This figure also clarifies how the API Gateway Central operates within a complex environment involving multiple agents, highlighting the comparison between the versions using Spring and Quarkus frameworks.

The architecture is designed to ensure resilience and fault tolerance by allowing each microservice to handle failures independently without affecting the entire system. For instance, if the Data Analysis Service encounters an error, it will not impact the functioning of the Data Collection or Alert Management Services. This separation of concerns ensures that each service can recover from failures without causing a system-wide outage. Furthermore, the use of stateless microservices enhances scalability. Stateless services do not store session information between requests, allowing them to handle many concurrent requests efficiently. By scaling horizontally, the system can add more service instances during high-demand periods, thus managing concurrent requests and reducing complexity. This is facilitated by containerization technologies like Docker and orchestration tools like Kubernetes, which automatically manage service deployment, scaling, and load balancing.

Microservices also provide a modular approach that simplifies the continuous enhancement of spyware detection and prediction capabilities. Each service can be independently updated and improved, allowing the system to adapt to new spyware types and emerging threats swiftly. This adaptability is crucial for maintaining an effective defense against cyber threats, as it enables the rapid deployment of updates and enhancements in response to changes in the threat landscape. By enabling the independent evolution of services, the system can remain agile and responsive, ensuring that new threats are addressed promptly and effectively.

**Figure 2:**
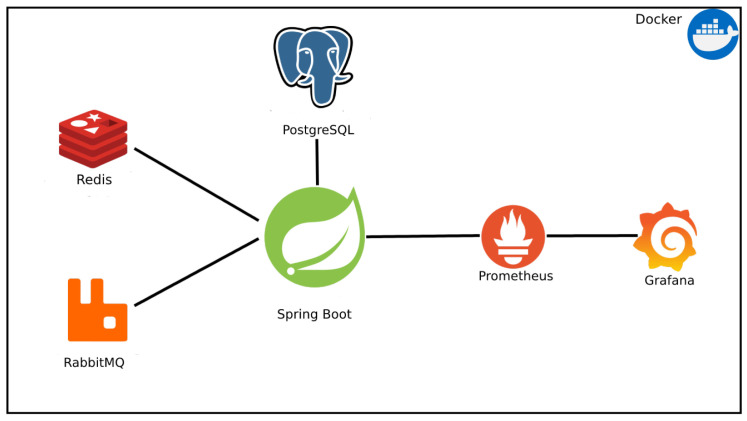
Architecture of the Central API Gateway.

Therefore, the focus of this article will be the Central API Gateway, whose architecture is presented in Figure 2. This architecture consists of some other components that will not be addressed in the tests and are presented in Figure 1, but here is a description of each:

- Admin: Platform administrator who will have access to Alerts through the Front-End application, being able to manage (remove and view) in addition to adding the monitoring management data;
- Front-End App: Application responsible for creating a secure, easy-to-use, and simple interface for the Administrator to manage the Application;
- User Database: Relational database to keep Front-End users separate from the rest of the application. The database will contain only one table to set up the login/registration of users, with encrypted passwords;
- Central API Gateway: This component will be responsible for centralizing the Alerts data and distributing them to the Front-End, with JSON Web Tokens (JWT), to ensure the security and reliability of the data, in addition to caching for fast data access and the messaging service to guarantee the delivery of Alerts;
- Alerts Database: Main relational database (PostgreSQL), responsible for maintaining the management data of monitoring and Alerts;
- Spyware: Main application component that will monitor the accessed sites, typed words, running processes, and typed hate speech and have a Port Scanner to assess if there are vulnerabilities on the PC. When any of these items are identified, the Spyware will generate an Alert and perform the capture of the information for sending to the API Gateway;
- API Gateway Spyware: The component that will contain an endpoint to communicate with the prediction model that will return whether a phrase is or is not hate speech;
- Prediction Model: Responsible for receiving a phrase in one of the languages (Spanish, Portuguese, or English), detecting the language, and processing through three multi-layer models, returning whether the phrase is hate speech. The model will be compiled with the Pickle library and inserted into the API Gateway.

The analyzed API uses some technologies to improve performance and observability and facilitate deployment in different environments; all technologies are presented in Figure 2. In this figure, it is possible to see that the web service is isolated in a Docker image along with a PostgreSQL database, used to store long-life data, a Redis database to store short-lived cache data, a RabbitMQ messaging service for managing processing queues, and two services for extracting and visualizing metrics, Prometheus and Grafana.

### 4.1. Improvements Applied to the Solution in Spring

Some structural and development measures were taken to improve the Spring application’s performance. The criteria that were changed and added to improve performance and maintain continuous delivery of the solution are as follows:

- Cache with Redis: To not overload the SQL (Structured Query Language) database with repeated and constant searches;
- Messaging Service with RabbitMQ: To maintain continuous updating and delivery of functionalities asynchronously;
- Initialization in “Lazy” Mode: The application Spring’s startup mode is changed to “lazy”, where only necessary components and dependencies are loaded;
- Exclusion of auto-configurations: Disabling automatic configurations of Spring not to consume resources unnecessarily;
- Switch of the Standard Servlet Container of Spring: The migration was made from Tomcat to Undertow, which showed better performance for Spring applications [46];
- Disabling Java Management Extensions (JMX): The flag for real-time bean monitoring was disabled to reduce unnecessary resource use, as other metric tools are being used;
- Removing the Standard Log System of Hibernate and Java Persistence API (JPA): Turning off database logs and creating controlled logs makes processing faster;
- Generating Indexes Sequentially: It is preferable in terms of performance due to its storage efficiency, better cache utilization, reduced fragmentation, and ease in ordered queries;
- Using Migrations for Database Table Creation: Replacing Hibernate’s automatic database structure creation with migrations allows for more refined control over schema changes, improving SQL database performance.

With these improvements, tests were conducted more efficiently, obtaining the results that will be presented later.

### 4.2. Improvements Applied to the Solution in Quarkus

Improvements were implemented in the Quarkus-developed application, including adopting strategies similar to those already applied in the Spring API. This involved configuration optimizations and programming, such as using cache and messaging strategies, adopting lazy loading mode, excluding non-essential autoconfiguration, and removing unnecessary logs.

In addition to the mentioned adaptations, other improvements were made to maximize performance in the Quarkus API. This included applying pre-compilation techniques to reduce startup time, using pooling strategies for database resources, minimizing memory use through efficient resource management, and implementing more granular and effective caching strategies for frequently accessed data.

### 4.3. Spyware

The development of the Spyware was carried out according to the established requirements and planned architecture. The Spyware was designed as a solution capable of tracking and monitoring elements identified as relevant to the specific context. The application implementation considered the previously defined architectural structure, ensuring proper integration with other system components.

Next, details on the implementation of the Spyware are presented, from the selection and implementation of monitoring techniques to the software’s integration with the architecture. The main technical and functional aspects are explored to provide a deep understanding of the operation and capabilities of this monitoring application.

#### 4.3.1. Monitoring

To achieve the defined objectives and requirements, the Spyware script had some monitoring features implemented through APIs and operating system libraries. Thus, the script runs in several threads, dividing into different specific functions:

- **Sniffer**: A network monitoring component that captures and analyzes packets in real time. This class operates in a separate thread and has attributes to store logs, captured DNS queries, and blocked sites. The main method, *sniffer()*, checks if a packet is a DNS packet with a payload and if the source port matches specific ports. If the DNS query matches a blocked site and has not been previously recorded, an alert is generated and added to the log list. Packet capture occurs in the *run()* method using the Scapy library, and packet processing is performed in the *process_packet()* method.
- **KeyLogger**: A class that records the user’s keystrokes. The class constructor initializes an empty log variable. The *callback()* method handles keypress events, checking if the key pressed is “enter” (to analyze the log and send an alert if necessary), “space” (to add a space to the log), “backspace” (to remove the last character from the log), or other keys, using the *get_shift_chars()* method to get special characters. The *report()* method checks if the log contains unwanted content, sending an alert if necessary and resetting the log. The *start()* method starts the KeyLogger.
- **Scanner**: Scans for open ports on a specified target. It uses the socket library to establish connections with the target’s ports and collect banners from the services running on those ports. During the scan, the open ports and banners are stored in lists. The class checks if any banner matches known vulnerabilities, generating an alert if a match is found.
- **ProcessLogger**: Identifies and handles malicious processes running on the system. The *are_malicious_process(self)* function checks for malicious processes by comparing the names of running processes with a list of malicious processes. If a malicious process is detected, it is terminated, and an alert is logged. The *get_process()* function retrieves an updated list of running processes.

#### 4.3.2. Captured Data

Capturing relevant information plays a fundamental role in analyzing the alerts generated by the spyware. The script can collect essential data, including running processes, the computer’s MAC address, screen images at the time of the alert, and the reason for the alert.

- **Running Processes**: Obtained by the *get_process()* function, which returns a list of running processes on the system.
- **MAC Address**: Obtained using the *get_mac_address* library in Python, which retrieves the MAC address of the system’s network interface.
- **Screen Images**: Captured using the *ImageGrab* library, which takes a screenshot of the screen and saves it in a buffer. The buffer is then encoded in base64 for storage and later analysis.

The reason for the alert is determined by the implemented monitoring, which checks for suspicious activities such as accessing blocked sites, malicious DNS queries, and the use of unwanted keywords, among others. These monitoring features allow for identifying the specific action that triggered the alert.

#### 4.3.3. Updating the Blacklists in the Central API Gateway

During the monitoring and alert generation process, the script analyzes the monitored points (DNS, processes, open ports, and typed words). It compares them with files containing lists of items that should not appear. These files are updated with data defined by the administrator in the Front-End application, which saves the information in the Central API Gateway.

The *update_aux_data()* function updates the auxiliary data used by the program. This process involves obtaining data from different categories (improper language words, vulnerable ports, malicious processes, blocked sites) through requests to a local API. The function stores the obtained data in text files, always updating the lists.

#### 4.3.4. Integration with the Prediction Models

After configuring the blacklists, implementing the monitoring, and capturing the necessary data, addressing the detection of hate speech is the final point considered. The KeyLogger captures sentences typed by the user, which are sent to the Prediction Models API Gateway to determine if they constitute hate speech.

The request is made via HTTP, using the POST method and a JSON-formatted body containing the phrase to be evaluated. The API Gateway response indicates if the phrase is classified as hate speech. If so, an alert is generated, thus integrating the detection of hate speech into the spyware’s operation and allowing an effective response to suspicious activities.

### 4.4. Prediction Model

The development of the solution includes a fundamental module responsible for using prediction models to detect offensive content in three different languages. A total of nine models were trained, with three for each specific language. To ensure the effectiveness and accuracy of the models, two datasets were chosen for each language, totaling six. For Portuguese, the datasets “Linguistic Datasets for Portuguese” [47] and “BraSNAM2018 Dataset” were selected. For Spanish, the datasets “Overview of the Task on Automatic Misogyny Identification at IberEval 2018” and “Overview of MEX-A3T at IberEval 2018” [48] were used. For English, the datasets “Improved Cyberbullying Detection Using Gender Information” [49] and “Are You a Racist or Am I Seeing Things?” [50] were chosen. These datasets were selected based on the availability of many phrases from real environments such as Twitter, Facebook, and YouTube.

Normalization and vectorization techniques were used to prepare the data for training. Using the NLTK library, texts were normalized by removing special characters, links, and mentions, converting them to lowercase, and applying stemming. Vectorization transformed each text into a vector containing word occurrence counts using the vectorizeText function, allowing the prediction models to identify patterns efficiently [51].

Nine models were selected: Logistic Regression, Support Vector Machine (SVM), and Multinomial Naive Bayes for each language. These techniques were chosen for their effectiveness in text classification and proven results in detecting hate speech [52]. Logistic Regression is ideal for multiclass classification [53], SVM is useful for non-linearly separable data [54], and Multinomial Naive Bayes is effective in capturing word frequencies [55].

Various techniques from the Scikit-learn library were applied to optimize the models’ performance [56]. Logistic Regression used the L1 penalty and the “saga” solver, with an inverse regularization strength parameter (C) of 1.2. SVM applied the “rbf” kernel, adjusting C and gamma parameters. Multinomial Naive Bayes used Laplace smoothing (alpha) and fitprior for estimating a priori probabilities. These strategies aimed to improve accuracy and generalization, avoiding overfitting and underfitting [57]; other definitions involving scenarios, adaptations, and security mechanisms will be used in the work described in [58,59,60]. Combining these models allows for effective hate detection in multilingual texts, providing a robust solution for text analysis.

## 5. Methodology

This chapter will discuss the tests conducted and the results obtained. It will be divided into specific tests of the Spring application and specific tests of the Quarkus application, and a comparison will be made between them, considering before and after the performance improvements were applied. All tests were performed on a computer with an i5 7200 CPU, 16 GB of RAM, and 1 TB SSD running the Kali Linux operating system.

Before the performance tests, simple tests were performed on all endpoints using Postman. These tests aimed to verify the functionalities and ensure the API’s proper operation. Then, JMeter was used to conduct more in-depth performance tests. Twenty-five tests were performed with different configurations of parallel request groups. Each set of requests consisted of three requests: one to obtain the authentication token, another to register an image, and another to register an alert.

The first request was a POST method to the endpoint ‘/login’, where the request body on the Listing 1 in JavaScript Object Notation (JSON) format was sent.

**Listing 1.** Login Payload.

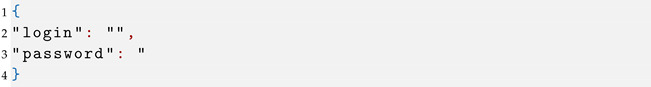



The previous request’s response provides a token for the next two requests. The next step is to make a POST request to save an image in the API. This request is sent to the endpoint ‘/image/save’ and must include the token obtained in the previous step in the headers. The request body must be in the JSON format as in Listing 2.

**Listing 2.** Image Payload.

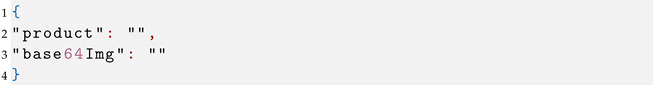



After the success of this request, the complete Image object containing an ID is returned, which is used in the next POST request to save the Alert. This request is sent to the endpoint ‘/alert/save’ and must include the token in the headers. The request body must be in the JSON format as in Listing 3.

**Listing 3.** Alert Payload.

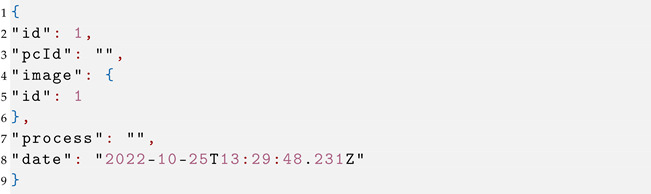



This set of requests was sent in different quantities in each test, varying in 500, 1000, 2000, 5000, and 10,000 parallel sends. However, when many requests were used, the JMeter’s memory heap would exceed, mainly due to sending images in base64 format in the request body. For this reason, a parallel application called API Tester was used to continue with more significant numbers of requests.

This set of requests was sent in different quantities in each test, varying in 500, 1000, 2000, 5000, and 10,000 parallel sends. The tests were conducted over different time intervals, such as over one hour, one day, and up to seven days, noting no significant difference in results over these periods. The average results, which will be presented forward, were obtained from these various time frames. However, when many requests were used, the JMeter’s memory heap would exceed, mainly due to sending images in base64 format in the request body. For this reason, a parallel application called API Tester was used to continue with more significant numbers of requests.

It is important to emphasize that the analyzed metrics were collected from the Docker image, which implies a minimum limit of memory and processing use. This limit is related to applications such as Grafana, Prometheus, PostgreSQL, Redis, and RabbitMQ. In natural environments, with more users and on more robust machines, resource use is expected to be dispersed, making the percentage of use of these applications negligible.

All applications, test plans, and raw results can be found in the project repositories, where it is also possible to view the tests before and after the performance improvements, in addition to more operational details [61,62,63,64,65].

### 5.1. Spring Application Results

**Figure 3:**
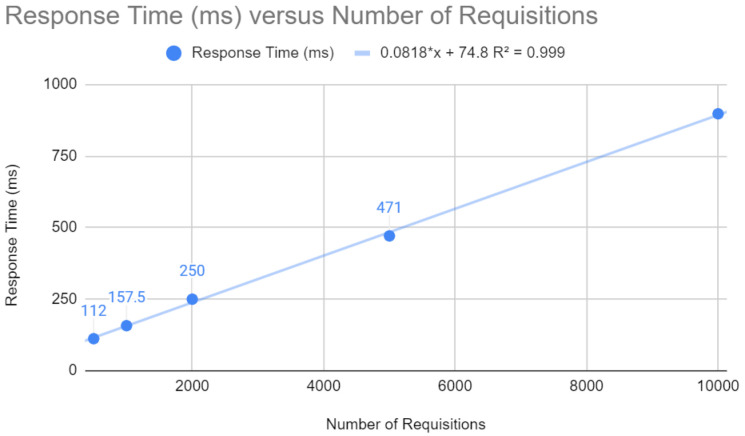
Testing requests with Spring.

Initially, some points were analyzed for the Spring application, which can be examined in the following figures. As illustrated in Figure 3, the response time in milliseconds increases with the number of requests, according to the equation y=0.0818x+74.8. Higher response times can result in a poor user experience, which emphasizes the importance of optimizations for fast response times in web applications.

**Figure 4:**
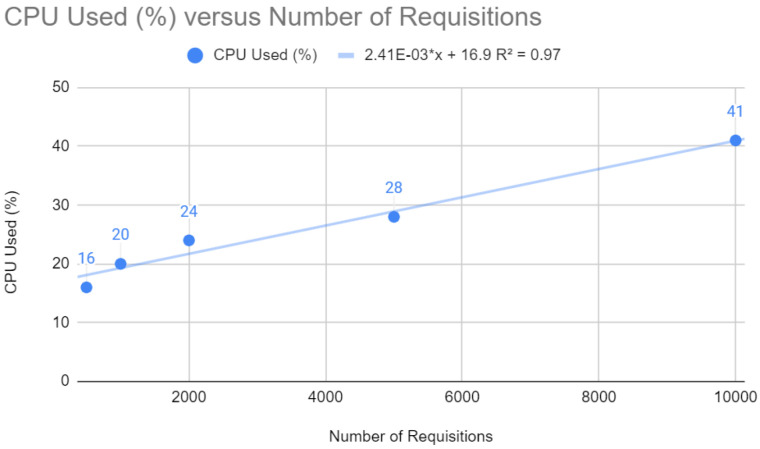
CPU testing with Spring.

Figure 4 depicts a linear relationship between the Central Processing Unit (CPU) usage percentage and the number of requests. The trend line equation is $y=2.41\times 10^{-3}x+16.9$, indicating that for each additional request, there is a corresponding increase in CPU usage of approximately 0.00241%. In web development, a linear increase in CPU utilization concerning the number of requests, as shown by the ratio, may suggest that the application is scaling as expected regarding workload. However, high CPU usage can also indicate that the application may not be efficient in terms of computing or that the server may reach its limit under heavy loads, which could lead to a degradation in performance or even failures.

**Figure 5:**
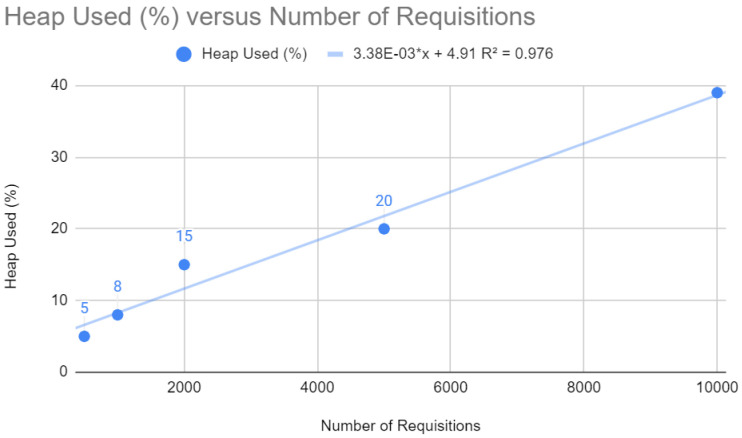
Heap testing with Spring.

Figure 5 shows the percentage of heap memory used as the number of requests increases, following a linear trend ($y=3.38\times 10^{-3}x+4.91$). The linear relationship shows that heap utilization increases with the number of requests. This is expected in web applications, as each request can create new objects. However, if heap memory approaches its maximum capacity, the system may face more frequent garbage collection problems, which can cause service pauses and affect application latency.

**Figure 6:**
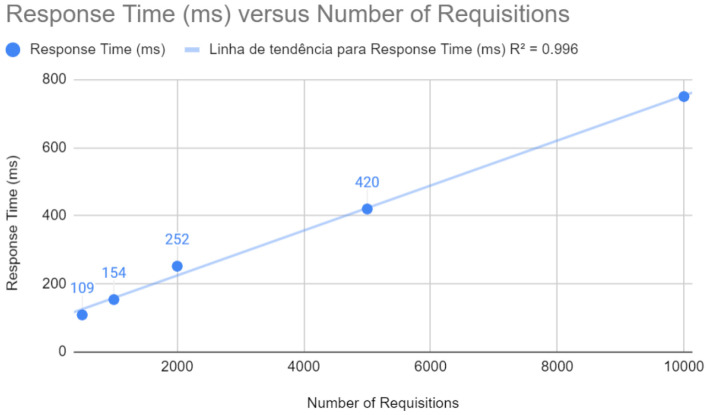
Requisition testing with Spring after improvements.

Below are the results after the improvements have been applied. Figure 6 shows that the response time maintains a remarkable linearity concerning the number of requests, with an $R^{2}$ of 0.996. This reflects the effectiveness of the improvements in ensuring that the application maintains consistent performance in terms of response time, a crucial factor for the end-user experience.

**Figure 7:**
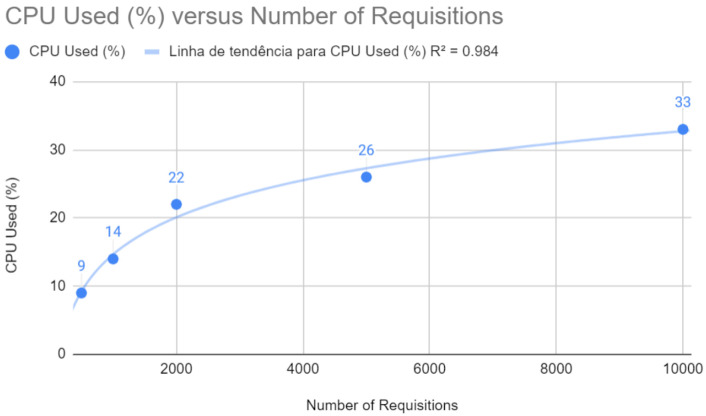
CPU test with Spring after improvements.

Figure 7 indicates a highly linear relationship between CPU usage and the number of requests with a coefficient of determination of $R^{2}=0.984$. This implies that the optimized application uses CPU resources more efficiently, an indicator of scalability and stability under increasing loads.

**Figure 8:**
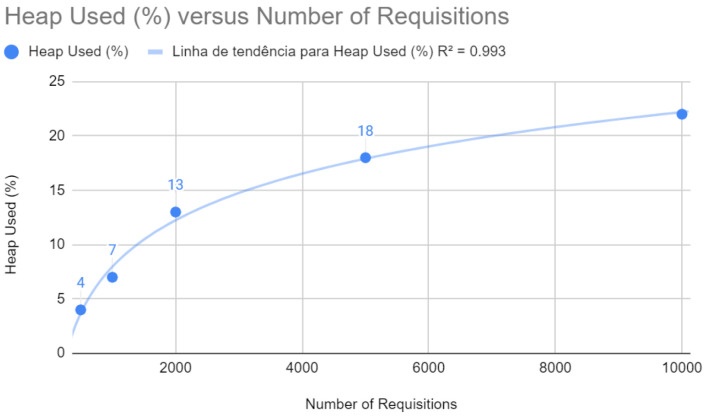
Heap test with Spring after improvements.

As illustrated in Figure 8, heap memory usage follows a linear trend with an $R^{2}$ of 0.993. The optimizations implemented seem to have improved memory management, maintaining stability and performance even with the increase in the number of requests.

### 5.2. Quarkus Application Results

**Figure 9:**
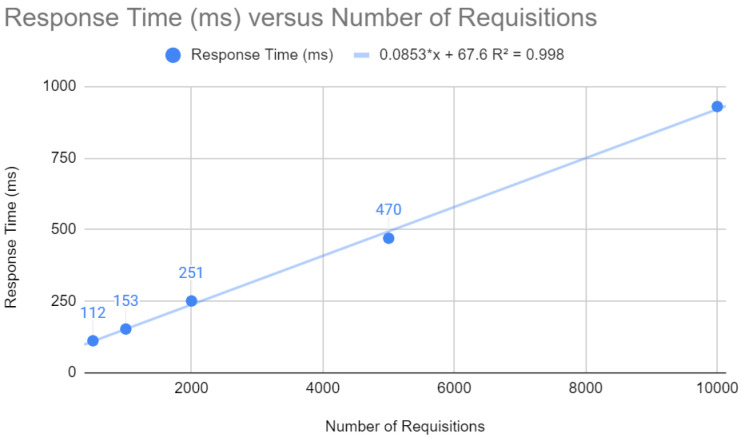
Testing requests with Quarkus.

The results of the Quarkus application are presented below. The response time concerning the number of requests, as shown in Figure 9, follows a linear trend y=0.0853x+67.6 with $R^{2}=0.998$. This indicates that for each additional request, the response time increases by a relatively small amount. In a web context, where fast response times are crucial, the Quarkus framework can keep latency low, even under a heavy load.

**Figure 10:**
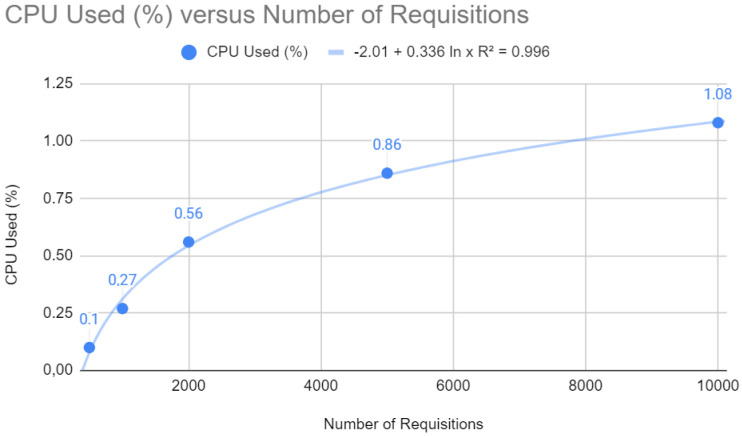
CPU test with Quarkus.

Figure 10 shows a logarithmic trend curve for CPU usage that stabilizes as the number of requests increases. The equation y=−2.01+0.336ln(x) with a coefficient of determination $R^{2}=0.996$ suggests that CPU usage grows initially, but the rate of growth slows down with more significant numbers of requests. Mathematically, this is a desirable characteristic, as it indicates that the application becomes less sensitive to peak demand as it scales, which indicates an efficient load distribution and good management of computing resources.

**Figure 11:**
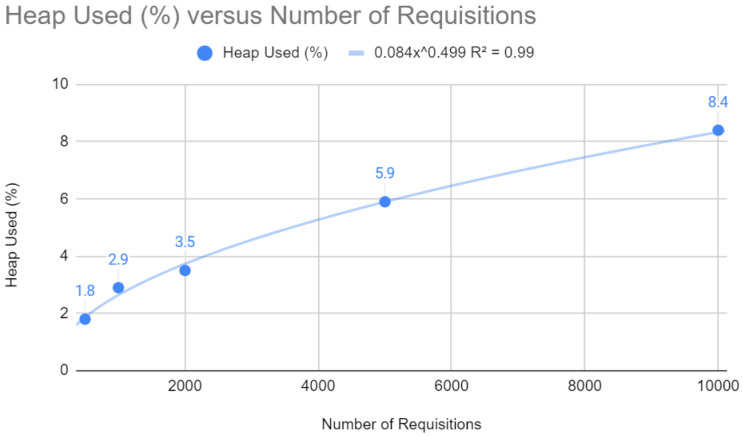
Heap test with Quarkus.

The relationship between heap memory usage and the number of requests, as shown in Figure 11, is described by a potential function $y=0.084x^{0.499}$ with an $R^{2}$ close to 1 (0.99). This shows that memory usage increases less proportionally than the number of requests, which implies efficient memory allocation and optimized garbage collection management, both of which are fundamental to the scalability of a web application.

**Figure 12:**
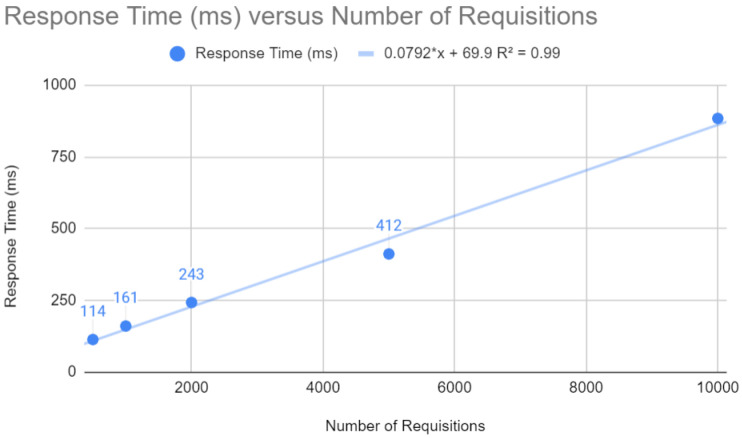
Request testing with Quarkus after improvements.

Differentiated results were obtained after the improvements were applied, as seen below. As shown in Figure 12, the response time increases linearly with the number of requests, indicating that the application maintains consistent performance even under high demand.

**Figure 13:**
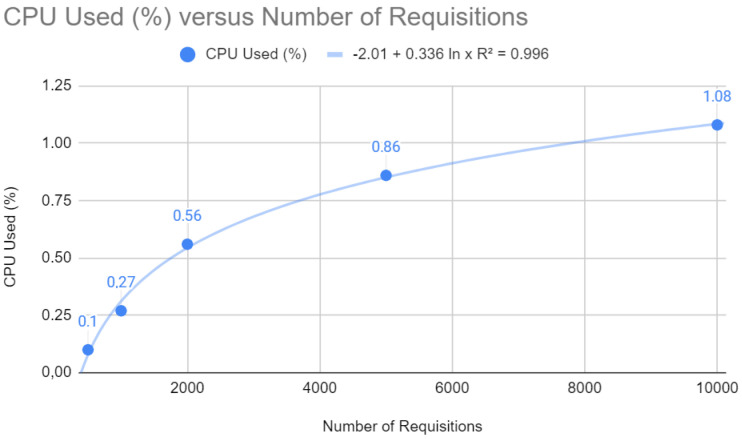
CPU test with Quarkus after improvements.

Comparing the previous results with the current ones, Figure 13 shows a significant reduction in the slope of the linear trend line of CPU usage. Mathematical analysis reveals that the optimized application now shows slower growth in CPU usage as the number of requests increases. This indicates that the application is scaling more efficiently from a computational point of view, as the additional load of each new request has a more minor impact on CPU usage.

**Figure 14:**
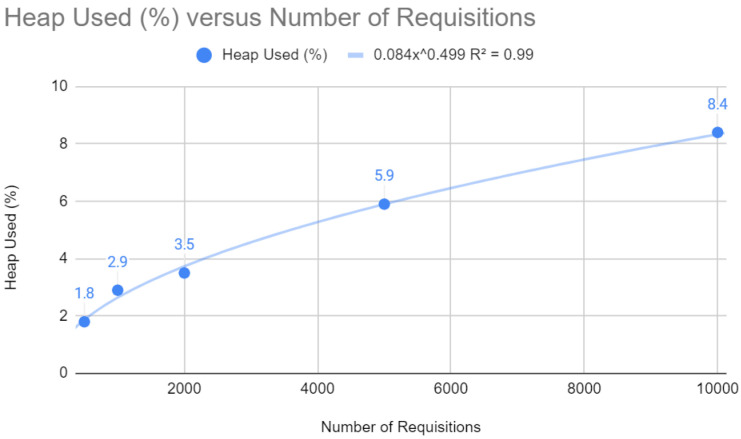
Heap test with Quarkus after improvements.

Looking at Figure 14, the heap memory usage curve after the optimizations indicates steeper asymptotic behavior than the previous results. The adjusted potential function suggests memory allocation and management efficiency, which is essential for web applications that operate with large amounts of data and require efficient memory management to avoid latencies and service interruptions.

### 5.3. Dimensioned Results Analysis

For different dimensions, mathematical formulas extracted from the empirical results presented previously were used. These predictions provide insights into how the application’s response time, CPU usage, and heap usage are expected to scale with increasing requests. For detailed numerical values, refer to Table 1, which illustrates the projected outcomes for 20,000 and 50,000 requests. The results underscore the need for optimization strategies to manage resource utilization effectively as the load increases.

Similarly, formulas from the Quarkus application results were used to examine the performance metrics designed for the respective application. The application demonstrates a different scaling behavior, particularly in CPU and heap memory utilization. The mathematical models used here indicate how efficiently Quarkus handles larger loads, with a lesser impact on CPU and memory resources. For specific values, including the expected response times and resource usage at 20,000 and 50,000 requests, see Table 2. These projections validate the Quarkus application’s robustness and efficiency in resource management under increasing operational demands.

### 5.4. Comparison between the Two Applications

The following section will present a meticulous comparison between the two frameworks. This analysis is anchored in empirical data obtained through performance tests conducted before and after applying specific optimizations. The metrics selected for this evaluation include the application startup time (uptime), CPU and heap memory usage percentage, and the response time to increasing requests. These metrics are fundamental to understanding the efficiency and effectiveness of frameworks in real production contexts.

**Figure 15:**
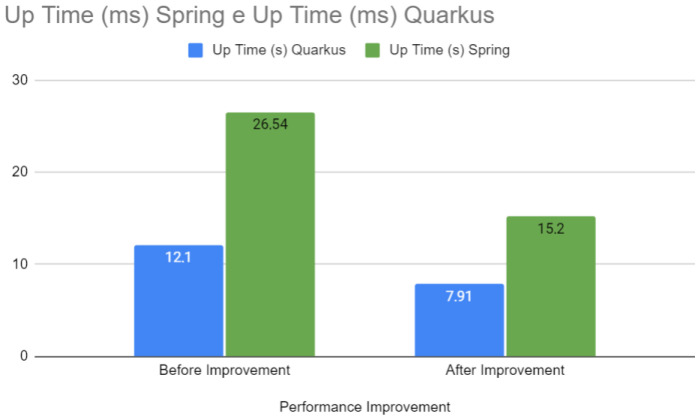
Uptime—Spring vs. Quarkus.

Figure 15 directly compares the uptimes before and after the optimizations for both Spring and Quarkus. The optimizations significantly improved the startup time for Quarkus, with a more modest reduction for Spring. In web development, startup time is a critical factor for the agility and responsiveness of services in production environments, especially in microservice-based architectures or when it is necessary to scale quickly to meet increased demand.

**Figure 16:**
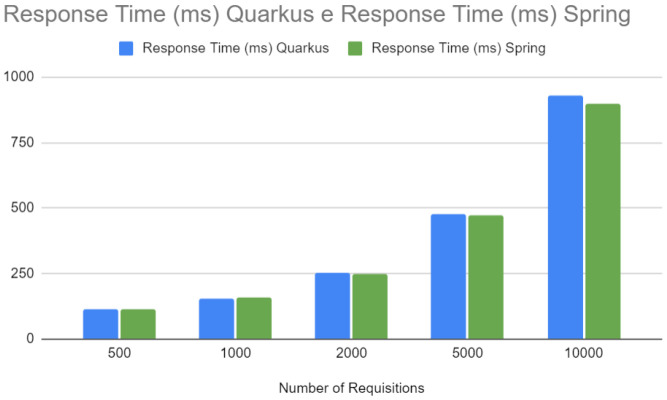
Requisition—Spring vs. Quarkus.

Figure 16 compares the response time between Quarkus and Spring. Although both frameworks show an increased response time with the number of requests, Spring shows a sharper increase. Response time is a critical indicator of user experience, and longer response times can result in a negative perception of the application. Fast and consistent response times are essential in production environments, especially for interactive or real-time applications.

**Figure 17:**
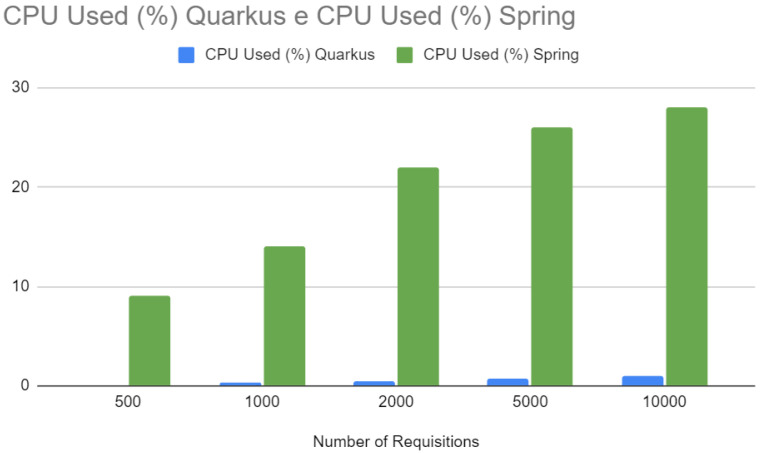
CPU—Spring vs. Quarkus.

Figure 17 compares the CPU usage between Quarkus and Spring at different numbers of requests. Quarkus consistently shows lower CPU usage across all data points. This reduced CPU usage indicates computational efficiency, which can reduce operating costs as it requires less computing power to perform the same amount of work. In addition, this could indicate that Quarkus may be better suited to environments where hardware resources are a concern, such as Internet of Things (IoT) devices or cloud computing environments where resource efficiency is essential.

**Figure 18:**
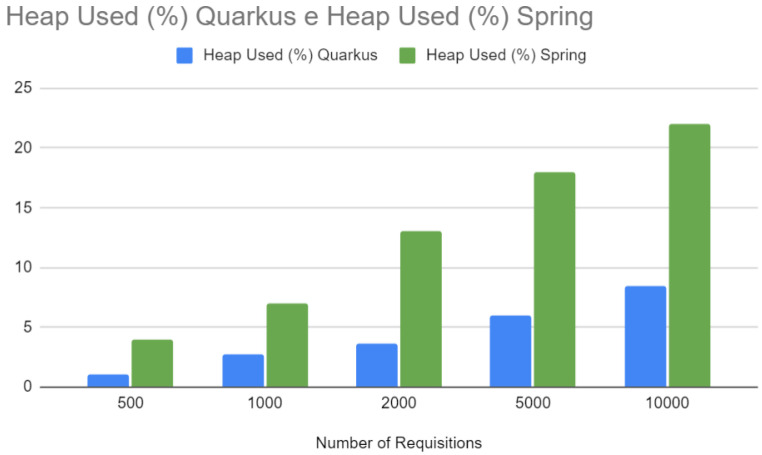
Heap—Spring vs. Quarkus.

Figure 18 compares heap memory usage. Like CPU usage, Quarkus demonstrates more efficient use of heap memory, which is particularly important in Java, where memory management can significantly impact application performance and latency. More efficient memory usage can result in less garbage collection and, therefore, more minor and less frequent pauses in application execution.

### 5.5. Prediction Models

The prediction models were evaluated using cross-validation and the Scikit-learn library. The data were split into training and validation sets using the train_test_split function. Three models were chosen: Logistic Regression, Multinomial Naive Bayes, and Support Vector Machine (SVM).

The evaluation metrics included the following:

- Accuracy
- Balanced accuracy
- Area under the Receiver Operating Characteristic (ROC) curve (roc_auc)

Cross-validation with 10 folds was used to ensure a reliable performance estimate. The trained models were saved with Pickle for later use. The results were visualized with graphs showing the variation in metrics, training time, and prediction accuracy.

#### 5.5.1. Accuracy

**Figure 19:**
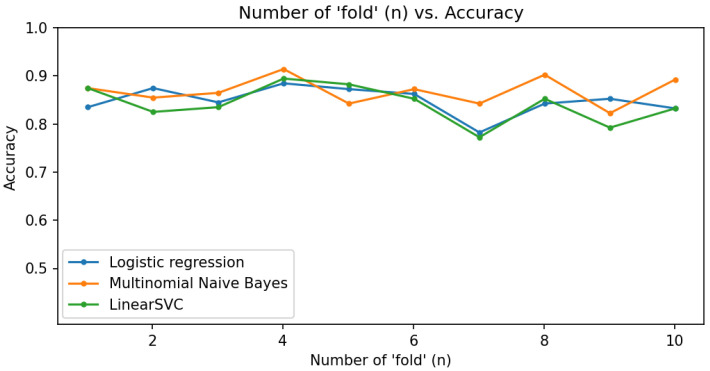
Accuracy graph.

The graph in Figure 19 shows the accuracy about the number of folds. The models presented an average of approximately 87% accuracy, with Multinomial Naive Bayes standing out slightly. This model’s robustness in handling text data likely contributed to its superior performance in detecting hate speech.

#### 5.5.2. Balanced Accuracy

**Figure 20:**
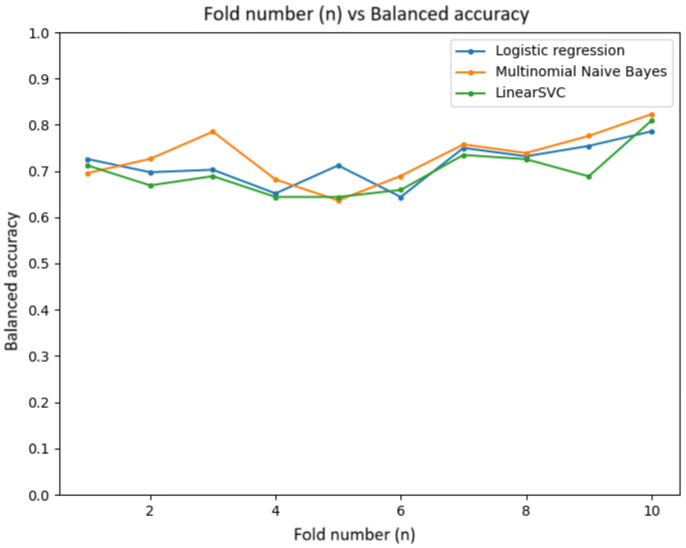
Balanced accuracy graph.

The graph in Figure 20 shows the balanced accuracy, which averaged 85%. This metric is important for unbalanced datasets. This indicates that the models could handle unbalanced data effectively, making accurate predictions across both classes.

#### 5.5.3. Area under the Receiver Operating Characteristic Curve (ROC-AUC)

**Figure 21:**
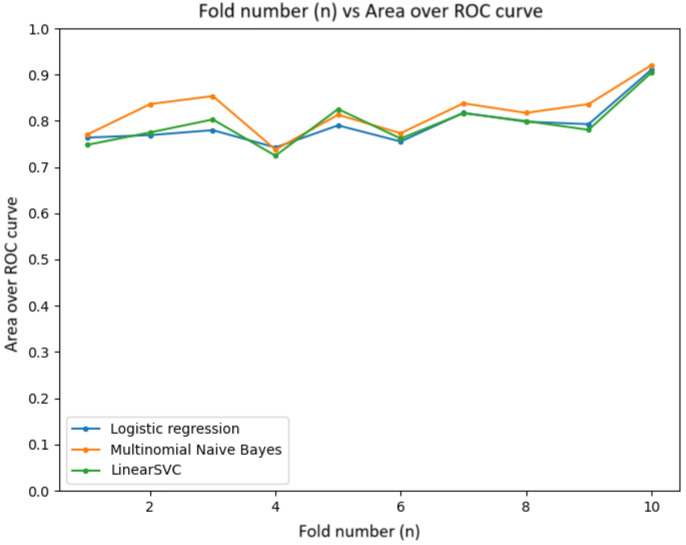
ROC-AUC curve graph.

The graph in Figure 21 illustrates the ROC-AUC curve, with average AUC values around 0.92. The models demonstrate a high ability to classify instances correctly.

## 6. Conclusions and Future Work

This study integrated spyware techniques and prediction models for efficient computer monitoring. However, it is crucial to recognize the ethical and privacy limitations related to the use of spyware. Future research should explore methods for balancing efficiency and privacy. Furthermore, developing more robust predictive models and applying these techniques in different contexts is recommended to validate their universality. In short, this study lays a solid foundation for future research and practical applications, highlighting the importance of continued advances in cybersecurity and systems monitoring.

It is crucial to emphasize that employees should be aware of the computer monitoring process, viewing it as a tool for learning and development rather than a punitive measure. The primary objective is to foster a positive work culture by discouraging hostile behaviors such as harassment, incivility, and intimidation.

Therefore, it is essential for companies to define unacceptable behaviors in employment contracts clearly. Additionally, implementing an effective feedback system for employees regarding the alerts generated by the monitoring system is necessary. This demonstrates the company’s commitment to maintaining a culture of diversity, equity, and inclusion, which positively impacts public perception and the business community’s view of the company’s reputation and corporate responsibilities. Furthermore, this approach plays a significant role in minimizing potential legal implications arising from inappropriate behaviors.

Several studies have consistently shown that Quarkus outperforms Spring Boot in various performance metrics, including startup time, memory usage, and response time. However, the specific context and configuration of the applications play a significant role in determining actual performance outcomes.

The studies reviewed highlight the superior performance of Quarkus in scenarios involving microservices applications. For instance, the research “A Performance Comparison of Spring Boot and Quarkus for Microservices” and “Performance Comparison of Spring Boot and Quarkus: A Case Study” consistently demonstrate Quarkus’s advantage in performance benchmarks. These findings underline the importance of context-specific evaluations when choosing a framework for application development.

The unique contribution of this study lies in its comprehensive approach, which includes the use of a complex application with extensive data and intricate relationships, as well as the application of rigorous statistical and mathematical analysis to benchmark evaluations. This thorough methodology provides valuable insights into the performance dynamics of both frameworks, offering practical guidance for developers and software architects.

In conclusion, while Quarkus often shows better performance metrics, the choice between Spring Boot and Quarkus should consider the specific requirements and context of the application. This study lays a robust foundation for future research and practical applications, emphasizing the importance of continuous advancements in cybersecurity and systems monitoring. Further research should continue to explore the balance between performance, scalability, and the unique needs of different computing environments to optimize the selection and use of development frameworks.

Overall, the findings of this study are crucial for advancing the understanding of performance in microservices architectures, contributing to more informed and effective technological choices in software development.

## Figures and Tables

**Table 1:** Predicted results for Spring application for 20,000 and 50,000 requests.

Test Description	Formula	20,000 Requests	50,000 Requests
Response Time (ms)	y=0.0818x+74.8	1685.6 ms	4168.0 ms
CPU Usage (%)	$y=2.41\times 10^{-3}x+16.9$	64.9%	137.5%
Heap Usage (%)	$y=3.38\times 10^{-3}x+4.91$	72.6%	175.9%

**Table 2:** Predicted results for Quarkus application for 20,000 and 50,000 requests.

Test Description	Formula	20,000 Requests	50,000 Requests
Response Time (ms)	y=0.0853x+67.6	1766.6 ms	4318.0 ms
CPU Usage (%)	y=−2.01+0.336ln(x)	7.2%	7.5%
Heap Usage (%)	$y=0.084x^{0.499}$	38.2%	56.4%

## Data Availability

Data is contained within the article.

## Abbreviations

The following abbreviations are used in this manuscript:

AMQP	Advanced Message Queuing Protocol
API	Application Programming Interface
CPU	Central Processing Unit
DB	Database
DBMS	Database Management System
DevOps	Development and Operations
DNS	Domain Name System
HTTP	Hypertext Transfer Protocol
IoT	Internet of Things
JMX	Java Management Extensions
JPA	Java Persistence API
JSON	JavaScript Object Notation
JWT	JSON Web Tokens
MQ	Message Queuing
MVCC	Multi-Version Concurrency Control
RAM	Random Access Memory
ROC	Receiver Operating Characteristic
ROC-AUC	Area Under the Receiver Operating Characteristic Curve
SQL	Structured Query Language
SVM	Support Vector Machine
